# Can zinc aluminate-titania composite be an alternative for alumina as microelectronic substrate?

**DOI:** 10.1038/srep40839

**Published:** 2017-01-13

**Authors:** Satheesh Babu Roshni, Mailadil Thomas Sebastian, Kuzhichalil Peethambharan Surendran

**Affiliations:** 1 Materials Science and Technology Division, National Institute for Interdisciplinary Science and Technology, CSIR, Trivandrum 695019, India; 2 Microelectronics Research Unit, Faculty of Information Technology and Electrical Engineering, University of Oulu, 90014, Finland

## Abstract

Alumina, thanks to its superior thermal and dielectric properties, has been the leading substrate over several decades, for power and microelectronics circuits. However, alumina lacks thermal stability since its temperature coefficient of resonant frequency (*τ*_*f*_) is far from zero (−60 ppmK^−1^). The present paper explores the potentiality of a ceramic composite 0.83ZnAl_2_O_4_-0.17TiO_2_ (in moles, abbreviated as ZAT) substrates for electronic applications over other commercially-used alumina-based substrates and synthesized using a non-aqueous tape casting method. The present substrate has *τ*_*f*_ of + 3.9 ppmK^−1^ and is a valuable addition to the group of thermo-stable substrates. The ZAT substrate shows a high thermal conductivity of 31.3 Wm^−1^K^−1^ (thermal conductivity of alumina is about 24.5 Wm^−1^K^−1^), along with promising mechanical, electrical and microwave dielectric properties comparable to that of alumina-based commercial substrates. Furthermore, the newly-developed substrate material shows exceptionally good thermal stability of dielectric constant, which cannot be met with any of the alumina-based HTCC substrates.

Low-loss ceramic dielectric substrates with high thermal conductivity have become an essential part of the electronic circuit boards for multichip power and microelectronic modules. The important prerequisites for a ceramic substrate for such applications include: (i) low relative permittivity (ε_r_ < 15), (ii) ultra low dielectric loss (tan δ < 10^−4^), (iii) high thermal conductivity (TC > 20 Wm^−1^K^−1^), (iv) low or matching coefficient of thermal expansion (CTE) to that of the materials attached to it (∼3–9 ppmK^−1^), and (v) temperature stable dielectric properties. Even though all the characteristics are equally critical, thermal conductivity has become increasingly important in the last two decades since heat generation density and resultant issues have escalated exorbitantly in much denser microelectronic circuits^1^,^2^. Due to the reason that ceramic materials possess interesting characteristics such as good chemical stability, high thermal conductivity, low magnitude of thermal expansion, high electrical resistivity, high mechanical strength etc., substrates such as Al_2_O_3_, AlN and BeO have been increasingly used for power electronic applications^3^,^4^,^5^. Among them, alumina (Al_2_O_3_) is the most widely used substrate because of its low cost, but in fact, its thermal conductivity is only about 24.5 Wm^−1^K^−1^ only. Aluminium nitride (AlN), though having high thermal conductivity (TC > 200 Wm^−1^K^−1^), is rather expensive due to the difficulty in synthesis under special atmospheric conditions. BeO, on the other hand, has good thermal performance (TC > 100 Wm^−1^K^−1^), but is being avoided by many manufacturers due to its toxicity when the powder is inhaled or ingested. Several alkali metal oxide free glass-ceramics have been reported to possess the ultra-low dielectric losses, which can be suggested as an alternative to alumina. However, in order to achieve high thermal conductivity the material must contain at least one alkali-ion containing phase, which aggravates thermal management issues^6^. More importantly, in applications where the thermal stability of the substrates matter, one has to look for alternative materials other than the three popular choices mentioned above, because all of them have higher temperature coefficients of resonant frequencies. Looking from an industrial perspective, temperature cycling reliability and mechanical stability in specific applications such as automotive, avionics and space are the driving force for the development of a new type substrate with promising thermal properties and low temperature coefficient of dielectric constant, *τ*_*ε*_.

Hence we undertake the challenging task of developing a novel high-temperature cofired ceramic (HTCC) with a rare combination of several characteristics of alumina, except thermal stability of dielectric constant, which alumina doesn’t have. In this work, we have employed tape casting technique for substrate development, since it is the best way to produce large thin flat ceramic sheets with controlled thickness, which cannot be produced by ordinary powder compaction, especially when cavities and vias are required in the substrates^7^. The tape casting procedure is more advantageous than conventional slip casting and gel casting methods also. The advantages include (i) easy control of the thickness of green sheet and also ability to laminate the green sheets to obtain required thickness, (ii) easy production of 2D or 3D ceramic parts with varying size, (iii) cost effectiveness and (iv) scope of mass production in a short period^8^,^9^.

Structurally, ZnAl_2_O_4_ is a member of spinel family and naturally occurring as mineral gahnite. In 2004, Van Der Laag *et al*. investigated the structural, elastic, thermo-physical and low-frequency dielectric properties of zinc aluminate^10^. The density of zinc aluminate tablets increases with sintering temperature and reached a maximum of 93% from 1300 °C onwards. A fully dense zinc aluminate, showed a Young’s modulus of 242 GPa, a dielectric constant of 10.6 and a thermal conductivity of about 20–25 Wm^−1^K^−1^.In 2004, Surendran *et al*.^11^ reported that ZnAl_2_O_4_ is an excellent candidate for electronic packaging applications due to its low permittivity and high Q_u_ × f value. The latter term, high Q_u_ × f (or low tanδ), is the typical figure of merit for a microwave dielectric, which is mandatory for high selectivity in microwave devices^12^. ZnAl_2_O_4_ has a relative permittivity of 8.5 with Q_u_ × f = 56300 GHz and *τ*_*f*_ = −79 ppmK^−1^. But the high negative *τ*_*f*_ (−79 ppmK^−1^) value of ZnAl_2_O_4_ restricts its use in practical applications. In the same report, Surendran *et al*. tailored the high negative *τ*_*f*_ of ZnAl_2_O_4_, close to zero by forming a composite with TiO_2_, which has a high positive *τ*_*f*_. The composite composition, 0.83ZnAl_2_O_4_ -0.17TiO_2_ (abbreviated as ZAT hereafter), showed nearly zero *τ*_*f*_ value with excellent microwave dielectric properties (ε_r_ = 12.67, Q_u_ = 9950 at 10.075 GHz)^11^. Later on, Surendran *et al*. also analyzed the microwave substrate characteristics such as the thermal conductivity, CTE, *τ*_*f*_, etc. of the bulk ZAT ceramics^13^. Apart from this, Lu *et al*. also investigated the effects of calcination temperature on the microstructures, phase compositions, and microwave dielectric properties of (1−x)ZnAl_2_O_4_–xTiO_2_ (x = 0.21). They reported that the secondary phase, Zn_2_Ti_3_O_8_, disappears completely at a calcination temperature of 1150 °C where the ceramics exhibits ε_r_ of 11.6, a Q_u_ × f of 74,000 GHz and a *τ*_*f*_ of-0.4 ppmK^−1^ 14. Lei *et al*. studied the microstructures, phase compositions and microwave dielectric properties of ZnAl_2_O_4_–TiO_2_ spinel-based composites.

The optimal microwave dielectric properties were attained in (1−x)ZnAl_2_O_4_–xTiO_2_ (x = 0.21) sintered at 1500 °C for 3 h with ε_r_ value of 11.4, Q × f value of 71,810 GHz (at 6.5 GHz), and *τ*_*f*_ of about −0.5 ppm/°C^15^. Interestingly in 2009, Huang *et al*. investigated properties of another composition, 0.5ZnAl_2_O_4_–0.5TiO_2_ ceramics as *τ*_*f*_ compensator for dielectrics. The sintering temperature of the specimen was reported to be effectively lowered to 1390 °C and ε_r_ of about 25.2 together with *τ*_*f*_ as large as 177 ppmK^−1^ was obtained. In order to verify its performance as a *τ*_*f*_ compensator, MgTiO_3_ and Mg_4_Nb_2_O_9_ dielectrics were individually mixed with as-prepared 0.5ZnAl_2_O_4_–0.5TiO_2_ to achieve a lower ε_r_, a high Q_u_ × f, and a nearly-zero *τ*_*f*_ 16. But so far, no serious investigations were reported on the tape casting of ZnAl_2_O_4_-TiO_2_ composites, which has every potential to compete with alumina-based microelectronic substrates.

In the present work, ceramic tapes of ZAT were developed using organic tape casting technique, and their microstructural, thermal, dielectric and mechanical properties were investigated. Alternatively, alumina was also tape cast under optimized conditions and their properties were compared to the newly developed ZAT substrate. In a systematically planned comparative analysis, we could reveal the striking similarity of ZAT with alumina substrates, with the former having superior thermal conductivity and thermal stability of the resonant frequency.

## Results and Discussion

### Properties of ZAT and alumina ceramics

**Figure 1:**
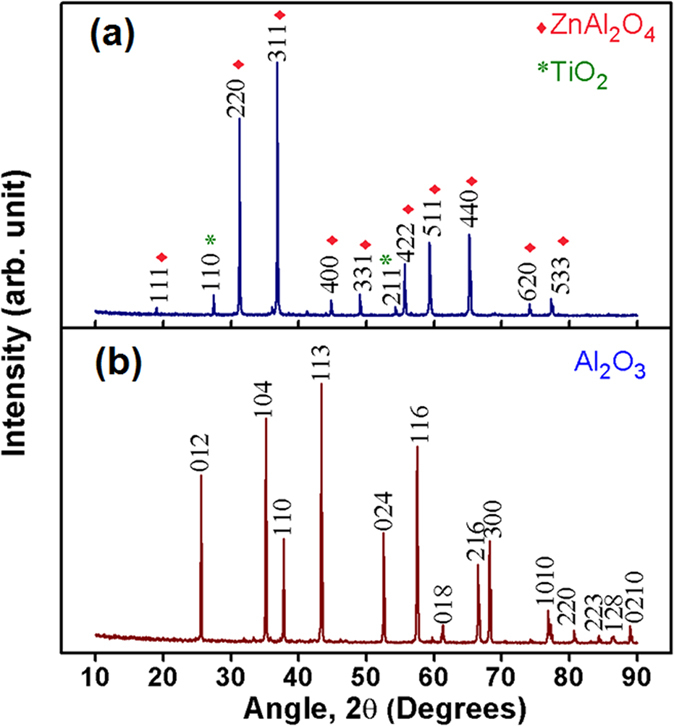
XRD pattern of (**a**) ZAT and (**b**) alumina.

Multilayer ceramic (MLC) substrates with high thermal conductivity are indispensably needed for interconnecting a semiconductor device to the next level of packaging. These substrates can accommodate wiring circuitry that provides for the transmission of signals and power to and from the semiconductor device, besides acting as a heat sink to cool the semiconductor device. The presence of any inorganic impurity or additional phase in the substrate may drastically affect the electrical performance of the substrate. It is interesting to note that the newly developed substrate material, zinc aluminate-titania composite, did not react among themselves to form any additional phase.This is clearly visible in Fig. 1(a), which is the X-ray diffraction pattern of the ZAT powder calcined at 1150 °C for 4 h. The contributing phases in ZAT were identified as the spinel zinc aluminate (Gahnite) with face-centered cubic system, having space group Fd3m and titanium oxide (rutile) with tetragonal symmetry having space group P4_2_/mnm. All diffraction peaks of zinc aluminate and titanium oxide (rutile) were indexed using JCPDS file no. 01-071-0968 and 01-072-1148 respectively. The XRD pattern of as-prepared alumina is indexed using JCPDS file no. 05-0712 is also given in Fig. 1(b).

The average particle size is considered as the critical characteristic required for the starting materials of tape casting slurry, since it controls the rheology, sintering temperature and density of the tape^17^. Even though smaller particles have an edge in terms of colloidal stability, ceramic powders with uniform particle size < 1000 nm can be qualified for making tape casting slip. In the present investigation, post calcined powder was ground and sieved to yield maximal uniformity in particle size distribution. The average particle size of ZAT is estimated as 589 nm whereas that of alumina is slightly large (793 nm).These values are satisfactory for making an optimal tape casting slurry.

### HTCC ZAT Ceramic Tape

#### Optimization of tape casting slurry

**Figure 2:**
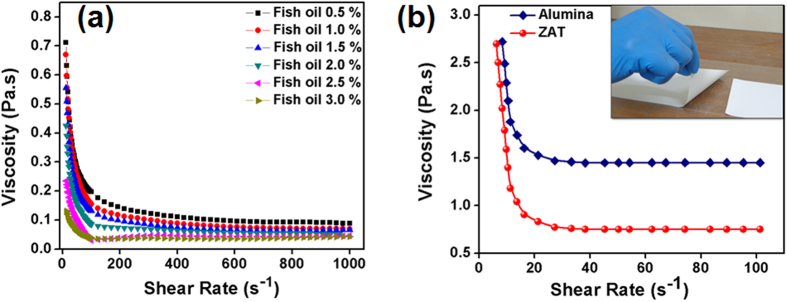
(**a**) Variation of viscosity with shear rate at different concentrations of the dispersant in ZAT slurry, (**b**) rheological behavior of the ready-to-cast ZAT and alumina slurry, (2 (**b**) inset) photo of a ZAT cast tape.

Currently, one of the well-accepted methods used for the manufacture of flat ceramic substrates with precisely controlled surface flatness is the tape casting technique. This versatile method requires an optimally formulated ceramic slurry, which can be cast to a flat sheet or layer, which is then dried into a flexible solid tape. The flow characteristics of the slurry were optimized using rheological measurements. For different wt. % of dispersant with respect to powder, the variation of viscosity as a function of shear rate is plotted in Fig. 2(a). From the figure, it is evident that viscosity decreases with increasing amount of fish oil up to 2.5% with respect to powder loading. In principle, a slurry with an optimal concentration of dispersant exhibits lower viscosity due to inter-particulate fluid layer coverage on each particle. Beyond the optimal concentration of the dispersant, the viscosity increases due to bridging flocculation^18^,^19^,^20^,^21^. By checking the stability against sedimentation, one can indirectly determine the optimum concentration of dispersant, which strongly affects the powder packing and sintering of cast tape. Obviously, particles of a well-deflocculated suspension settle rather slowly, as illustrated in Supplementary Fig. S1. It describes the colloidal stability of ZAT suspensions with fish oil as the dispersant for a range of concentrations (0.5 to 3.0% with respect to powder loading). In this study, the sediment height (*H*) was observed at regular intervals as a function of time against the initial height (*H*_o_) in a graduated cylinder of 10 ml volume. It should be noted that a well-deflocculated slip should have a slow settling rate and a much higher packed bed density than partially flocculated slurry^7^. As seen, ZAT slurry with 2.5% of dispersant with respect to ceramic powder shows a slower rate of sedimentation and higher packing density. From the viscosity analysis and sedimentation results, one can conclude that 2.5% of fish oil with respect to ceramic powder is enough to obtain a well-dispersed suspension. In the later stage, binder, plasticizers and homogenizer were also added to the slurry (see Table 1). The binders are long chain polymers, which are used in tape casting slurry in order to impart strength to the green tape after the evaporation of solvents, by forming organic bridges between ceramic particles. Plasticizers provide flexibility to the green tape which is classified as Type I, which softens the polymer chains of the binder molecule and Type II, which lubricate the slurry. The homogenizer retards the skin formation during drying of the tape. In order to obtain a flexible defect free green tape, the relative concentration of binder-plasticizer system should be suitably adjusted^7^,^22^. Figure 2(b) shows the viscosity analysis of ready-to-cast final slurry of ZAT as a function of shear rate. From the figure, it is clear that the slurry shows a pseudoplastic behavior (shear thinning behavior), which is highly desirable for a tape-casting slurry because it experiences a shear force under the action of the blade or moving carrier^23^. The considerable increase in the viscosity of final slurry is mainly contributed by the addition of binder in it. Inset figure in 2(b) is the image of a thin green tape; cast using the given ZAT composition. The viscosity analysis and the composition of the final tape-casting slurry of alumina are also provided in Fig. 2(b) and Table 1.

**Figure 3:**
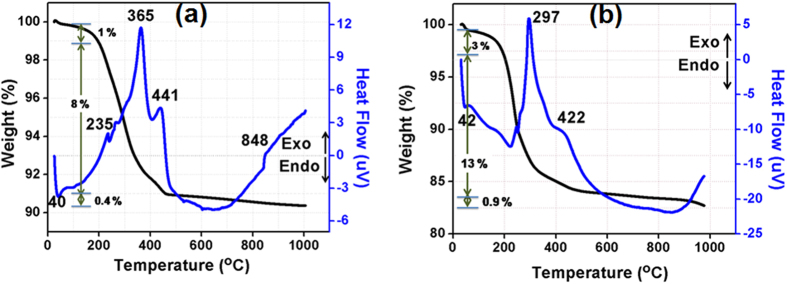
(**a**) TGA/DTA of ZAT tape and (**b**) TGA/DTA of alumina tape.

A clear understanding of the thermal decomposition of organic matter contained in the green tapes is essential to minimize the undesirable defects such as delamination, cracks, anisotropic shrinkage, and camber occurring during sintering. In order to understand the thermal evolution history of ZAT tapes, a combined thermogravimetric (TGA)/differential thermal (DTA) analysis was performed, whose results in terms of weight loss and heat flow as a function of temperature are shown in Fig. 3(a). TGA is done in air atmosphere up to a maximum temperature of 1000 °C with a heating rate of 10 °C/min. The endothermic trench down to 200 °C may be due to volatilization of the adsorbed water on the surface of the tape. As shown, a total weight loss of ∼8% is observed from 170 °C to 450 °C due to the burnout of organic additives. Of this, a rapid weight loss of around 6% observed up to 365 °C is mainly due to the decomposition of low molecular weight organics like dispersant and plasticizer. This is reflected in the exothermic peak around 365 °C, which is followed by a minor weight loss up to 450 °C consequent to the binder burnout (see the diffused peak at 450 °C in DTA curve). The TG curve shows that a negligible weight loss (~0.4%) above 600 °C as reflected in the slow ascending of exothermic curve, which was believed to be due to the gradual combustion of residual carbon^24^,^25^,^26^,^27^. For alumina, a total weight loss of ∼11% is observed from 190 °C to 550 °C as shown in Fig 3(b). The decomposition of organic additives is reflected in the exothermic peak around 297 °C. Based on the TG/DTA analysis in Fig 3(a) and (b), the sintering profile for ZAT and alumina tape are constructed as presented in Supplementary Fig. S2.

During the sintering process, anisotropic shrinkage is observed in most of the ceramic tapes, which can be an obvious consequence of the anisotropy in its microstructure, originated from casting process itself. This is because during casting, the slurry is sheared while passing under the doctor blade, resulting in a reorientation of the particles and binder molecules. As a result of the high viscosity and pseudoplastic nature of the slurry, this arrangement will be frozen in, immediately after casting. The post-cast drying is yet another contributing factor towards anisotropicity. This is because; due to capillary force, an increased amount of binder is pushed to the top surface of the tape, giving rise to particle rearrangement. Since the cast film adheres to the Mylar^®^(polyethylene terephthalate film) substrate, drying shrinkage will be in the z-direction^28^. The shrinkage of ZAT tape during sintering at 1500 °C is observed as 8%, 11% and 15% in x, y and z-direction respectively (see Supplementary Fig. S3). Alumina tape also exhibits a similar shrinkage upon sintering at 1600 °C.

#### Surface morphological studies of ZAT and alumina tape

**Figure 4:**
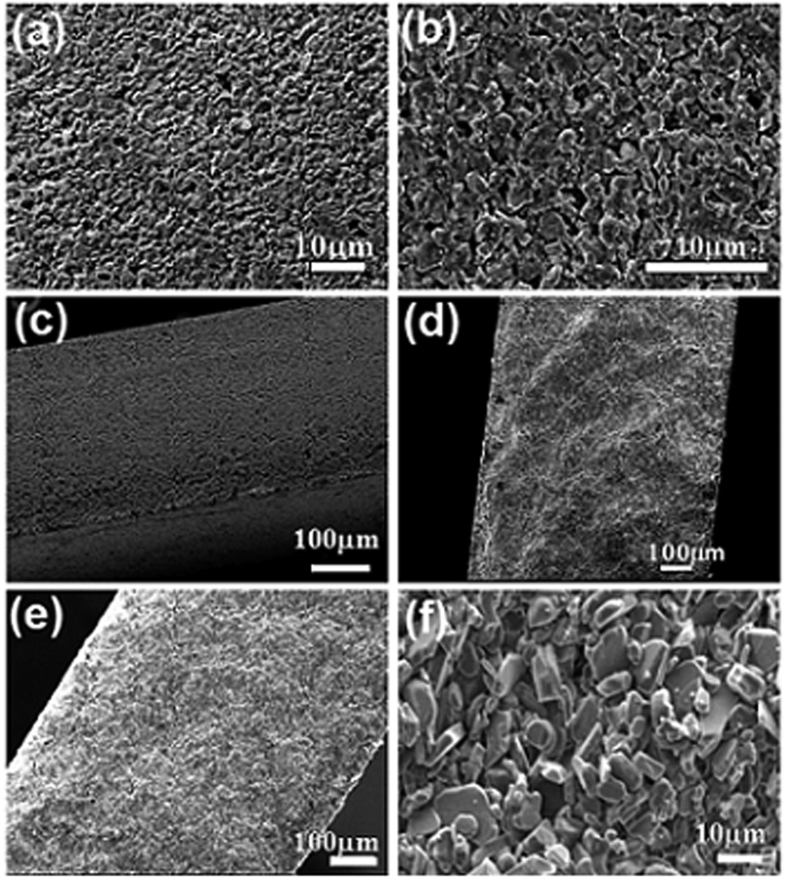
SEM images of the surface of ZAT (**a**) green tape, (**b**) sintered tape (fractured), (**c**) cross section of thermo-laminated stack (8 layers), (**d**) cross section of thermo-laminated multilayer stack after sintering and (**e**) cross section and (**f**) surface of alumina thermo-laminated multilayer stack after sintering.

A dense and homogeneous microstructure with low content of porosity is observed in the micrograph image of ZAT green body, which is given in Fig. 4(a). The denser microstructure is believed to be due to high solid loading and the optimal amount of organic additives. As shown in Fig. 4(b), which was recorded after sintering at 1500 °C, densification and grain growth occurred, resulting in slightly enlarged particles with fractional porosity. The cross-sectional morphology of thermo-laminated stack (8 layers of tape in the same batch) before and after sintering are shown in Fig. 4(c) and (d) respectively, which demonstrate that the laminate is defect-free with no sign of inter-layer boundaries and cracks. Furthermore, it also reveals that the thermo lamination conditions are ideal so that the individual layers merged into a homogenous coherent body during sintering. The SEM images of cross-section and surface of alumina tape are given in Fig. 4(e) and (f), which presents a homogenous microstructure. On sintering, alumina tapes presented a morphology showing relatively larger grains with the inclusion of fractional porosity. The RMS grain size of ZAT, estimated using line intercept method on typical scanning electron micrographs, is about 4 ± 1 μm while that for alumina is about 7 ± 2 μm. It should be noted that no sintering aids were used in both alumina and ZAT.

**Figure 5:**
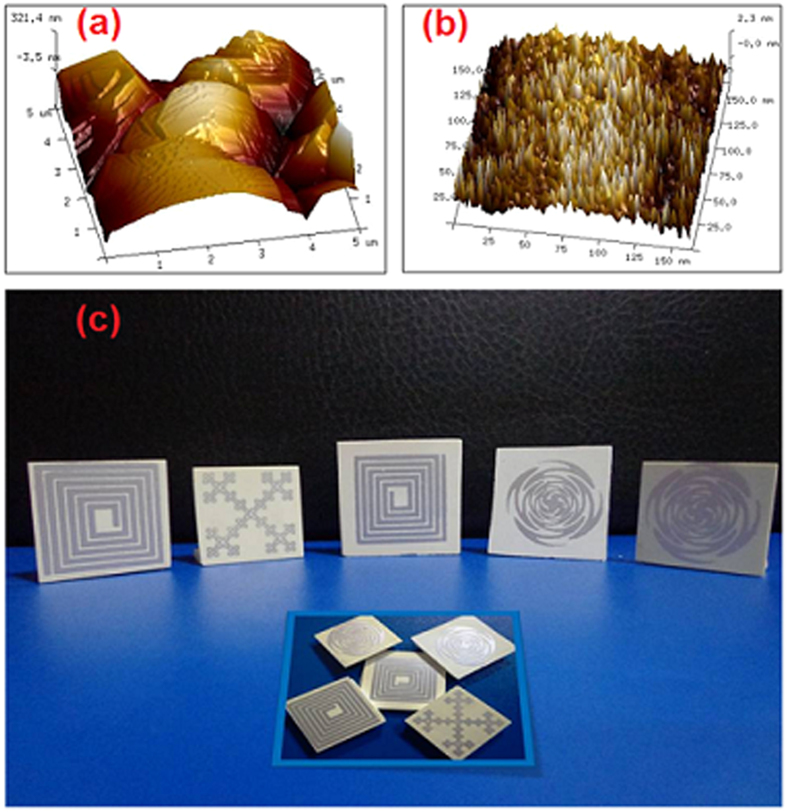
3D view of tapping mode AFM images of (**a**) unpolished and (**b**) polished ZAT sintered tape after sintering at 1500 °C and (**c**) Antenna patterns screen printed on sintered ZAT tapes.

The solderability of a conductor on the substrate largely depends, besides the oxidation of the conductor, on the surface roughness of the substrate^29^. The 3D topographic images of the sintered ZAT sample (both unpolished and polished) in tapping mode are shown in Fig. 5(a) and (b). The average roughness (R_a_) of the unpolished and polished sintered sample is about 85.4 nm and 4.7 nm respectively. Surface smoothness is highly desirable for substrate materials, which otherwise lead to increased high-frequency circuit loss^28^. A few predesigned antenna patterns screen printed using commercial Ag paste on polished ZAT substrates are shown in Fig. 5(c). These printed structures show high scratch resistance and good surface homogeneity suitable for antenna applications, whose radiation characteristics largely depend on the microstructural homogeneity and adhesion to the substrate.

### Mechanical properties of the tape

**Figure 6:**
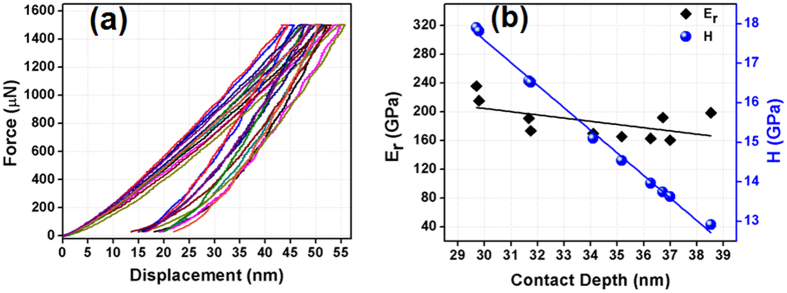
(**a**) Force vs. displacement graph and (**b**) Reduced modulus and hardness as a function of contact depthof ZAT ceramic tape.

In electronic packages, the ceramic substrates have to withstand the stress encountered in several occasions such as screen printing, isostatic lamination pressing, cofiring, post-sintering machining as well as during heating and cooling of the device. Therefore it is essential to understand the mechanical properties of the newly-developed substrate material. As an efficient tool to provide small volume mechanical characterization, nanoindentation is usually performed to obtain the hardness and reduced elastic modulus of thin ceramic sheets. Factors, which dictates the hardness of a material are its crystal structure, type of bonding among atoms, density, grain size and purity of the ceramic material^30^. In a typical nanoindentation test, a predetermined load (p) is applied to the indenter, which presses against the sample surface and the resultant depth of penetration (h) on the surface was measured^31^. The p-h curve with the indentation performed by identical loads at different locations of the specimen is depicted in Fig. 6(a). The loading and unloading profile of sintered ZAT sample with periodic loads ranging from 0 to 1500 μN, are recorded and analyzed as a function of depth of penetration. Closely inspecting Fig. 6(a), one can assume that ZAT ceramic substrate follows the behavior of mixed elastoplastic deformation. This behavior is usually due to the presence of elastic and plastic zones in the specimen close to indenter^32^,^33^.

By knowing the geometry of indenter and depth of penetration, the area of contact is calculated, from which the elastic modulus and hardness were estimated. The slope at initial stages of an unloading curve represents the elastic stiffness, S is given by


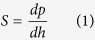


In the present nanoindentation testing of ZAT ceramic tape, cube-cornered indenter was used to assure precise control of indentation mainly due to the well-defined pointer that exerts higher stress and strain in the area of contact. For materials having high surface roughness, cube corner indenter provides better indent at peaks and valleys in order to produce more repeatable data compared to other indenters. Hardness or mean contact pressure of material is obtained from projected area of contact at maximum load^31^. Projected area of contact, A is given by,


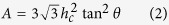


which can be approximated to 24.5h_c_ 2, where, h_c_ is depth of penetration and θ is the face angle for cube corner indenter, 34.3°. The hardness, H can be calculated as,


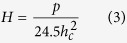


where p is the maximum load applied.

The reduced Young’s modulus of the specimen, E_r_ is calculated using the well-known relationship based on conventional Oliver Pharr method^34^,


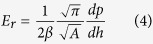


where β is a constant depending on the shape of the indenter and A is the area of the indentation^33^. The value of β for cube corner indenter is about 1.034. Obviously for harder materials, the depth of penetration is likely to be smaller. In the case of ZAT, the hardness and reduced modulus at a penetration depth of 29.7 nm were calculated to be 17.9 GPa and 235.8 GPa respectively (see Fig. 6(b)). Gradient and 3D *in situ* scanning probe microscopy (SPM) image of ZAT substrate after indentation using the same cube corner indenter are also shown in Supplementary Fig. S4, where the indentation imprints are marked in white circles. The average roughness (R_a_) obtained for ZAT tape at the test location is estimated to be 75.90 nm.

#### Thermal properties of ZAT and alumina tape

**Figure 7:**
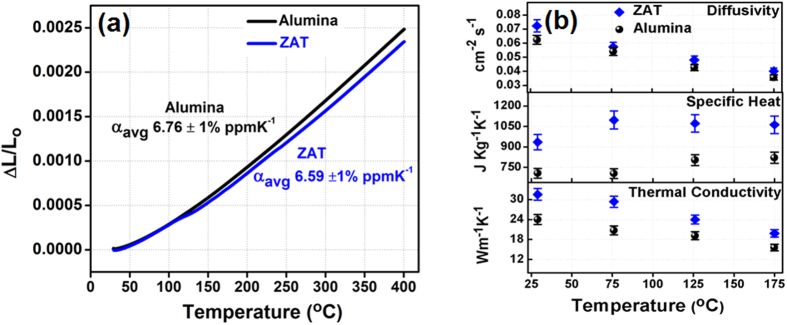
(**a**) Thermal expansion characteristics and (**b**) Temperature variation of thermal diffusivity, specific heat and thermal conductivity of ZAT and alumina.

CTE was assessed using thermomechanical analyzer that accurately measures the changes in length of sample when it is heated over a programmed temperature range (30 °C to 400 °C) as shown in Fig. 7(a). The linear CTE can be calculated as follows:


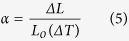


where α is the linear coefficient of thermal expansion, L_o_ is initial sample length at room temperature, ΔL and ΔT are change in length and temperature respectively. Figure 7(a) shows that, as temperature increases, the material shows a tendency to expand i.e. it exhibits a positive CTE, which is typical for most materials. This is mainly due to the increased kinetic energy which an atom gains at elevated temperature and, as a result, constituent atoms maintain a greater separation than at lower temperature. The graph shows that the apparent CTE value of ZAT is about 6.59 ± 1% ppmK^−1^ which is close to alumina (6.76 ± 1% ppmK^−1^). Ceramic materials have low CTE (0.5 to 15 ppmK^−1^) as compared to metals and polymers, due to their strong interatomic bonding forces (ionic and covalent)^35^. Ideally, the CTE of present ceramic substrate should be comparable with that of alumina and other substrates like AlN and BeO (4–9 ppmK^−1^)^36^,^37^. The low CTE value of ZAT qualifies it to be used successfully as a substrate in most electronic devices.

A dense microelectronic circuit with high processing speed is expected to generate more heat while in operation. One of the most economical ways to solve this problem is to develop ceramic substrates with high thermal conductivity that can dissipate heat rapidly from the circuit. Currently, there are a few high thermal conductivity materials such as beryllia, aluminium nitride etc. that can be used as heat sinks in electronic devices^38^. However, as mentioned in the introduction, these materials have genuine issues of toxicity and cost, which largely restrict their popularity. Our effort is to find an alternative to alumina, which is a moderate thermal conductor and inexpensive.

By knowing the specific heat and density, the thermal conductivity can be estimated by the following equation^39^


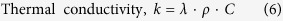


where, *λ* is the thermal diffusivity, *ρ* is the density of the material and *C* is the specific heat of the material. Since the sintered ceramic samples are not fully dense, the porosity correction has to be applied. The modified porosity corrected thermal conductivity was calculated as^40^,


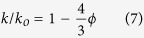


where, k_o_ and k are the porosity corrected and actual thermal conductivity values, *ϕ* is the fractional porosity of the ceramic and the coefficient 4/3 is included to remove the effect of porosity. The room-temperature thermal conductivity of ZAT and alumina ceramics are computed as 31.3 Wm^−1^K^−1^ and 24.5 Wm^−1^K^−1^ respectively. Evidently, the ZAT shows a relatively high thermal conductivity compared to alumina and other commercial HTCC substrates available in the market. Generally, high thermal conductivity of a typical ceramics can be correlated to have a number of interesting common features like simple crystal structure, low atomic mass, strong inter atomic forces, high atomic packing density, comparable atomic weight differences among the components in the composition etc.^41^. The thermal conductivity is in reciprocal relation with square root of mean atomic mass in the material. Interestingly, the elements in ZAT composition possess relatively low atomic weight. The melting temperature of ZAT ceramics is around 1600 °C 42, which can be an indirect indicator of strong interatomic bonding, a prerequisite for high thermal conductivity.

From the Fig. 7(b), it is clear that the thermal diffusivity and thermal conductivity decreases with increasing temperature, which is typical for polycrystalline ceramics. As per the theory of heat conduction^40^, the thermal conductivity is directly proportional to mean free path of phonons. As the temperature increases, the number of phonon collisions increases and hence the relative probability for high rate of scattering also increase. The high scattering of phonons in combination with other extrinsic factors such as lattice imperfections (impurities, dislocations, vacancies, porosity, grain boundaries, etc.) will result in a decreased mean free path. This, in turn, will reduce the thermal conductivity of ZAT to lower values with increasing temperature.

#### Electrical and dielectric properties of ZAT and alumina tape

**Figure 8:**
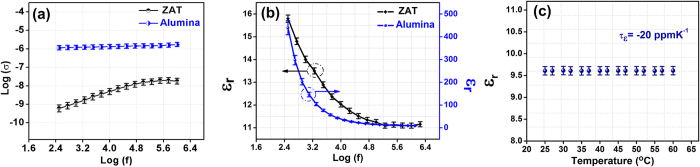
(**a**) Frequency dependent AC conductivities of alumina and ZAT ceramics, (**b**) Dielectric constant of bulk ZAT and alumina ceramics as a function of frequency, (**c**)Temperature dependence of dielectric constant of ZAT measured at 5 GHz.

Frequency dependent AC conductivity of ceramic materials is usually explained in terms of Jonscher’s universal power law^43^


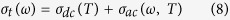


where, σ_t_ is the total electrical conductivity, σ_dc_ is the frequency independent DC conductivity contributed by the mobile electrons. In ceramics, σ_dc_ is very small compared to total conductivity. σ_ac_ is the frequency dependent electrical conductivity, which is given by the power law^43^


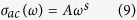


where, constant A represents the strength of polarizability and exponent ‘s’ indicates the degree of interaction between mobile ions and lattice around them. In the Fig. 8(a), as frequency increases, a small increase in AC conductivity is observed. When the frequency of applied field is increased, the conductive grains become more active by boosting the hopping between ions. The frequency at which electrons start hopping between ions is called hopping frequency. As the frequency increases, the hopping frequency increases and a gradual increase in conductivity with frequency is observed^44^. With increasing frequency in ZAT dielectrics, the hopping frequency also increases resulting in a gradual increase in conductivity. On the other hand, alumina follows a nearly constant loss behavior which corresponds to S = 1 in equation 9, where the dielectric loss is likely to be independent of frequency^45^.

The frequency dependence of dielectric constant of bulk ZAT and alumina ceramics at radio-frequency (300 Hz to 3 MHz) at room temperature is provided in Fig. 8(b). It is a well-known fact that the polarization of a dielectric material contributes to the dielectric constant. At lower radio frequencies, all the four polarization mechanisms (electronic, dipolar, ionic and interfacial) will come into effect with contribution from the interfacial polarization at the highest, resulting in high dielectric constant. As the frequency increases, the influence of dipolar polarization will take over interfacial polarization, which can be observed as a reduction in the dielectric constant^46^,^47^. Since sintered ceramic bodies are likely to contain a finite amount of porosity, which can detrimentally affect the apparent dielectric properties, Penn *et al*. arrived at an empirical relation for the porosity compensation of dielectric constant as given below^48^:


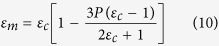


where ε_m_ and ε_c_ are the measured and corrected dielectric constant, P is the fractional porosity. Using this relation, the dielectric constant, ε_c_ of ZAT and alumina ceramics are computed to be 11.2 and 9.2 respectively at 1 MHz.

For the stable operation of any electronic circuits, the properties of substrates such as resonant frequency, dielectric constant, etc. should not change with an external variable such as temperature. Since circuit heating is very likely in multilayer modules, information pertaining to the thermal variation of the dielectric constant is very critical. The temperature coefficient of dielectric constant is obtained from the graph using the relation^12^,


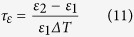


where, ε_1_ and ε_2_ are the dielectric constant at two different temperatures, ΔT is the corresponding temperature difference.

The relationship between *τ*_*f*_ and *τ*_*ε*_ is given by:


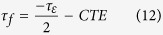


As shown in Fig 8(c), the variation of dielectric constant with respect to temperature is constant in the range of 25–65 °C. It must be recalled that the value of *τ*_*f*_ of alumina is −60 ppmK^−1^ 12, which is far from being thermo-stable while for the present substrate ZAT, it is + 3.9 ppmK^−1^, much closer to zero.

The dielectric strength of a material is defined as the maximum electric field that a material can withstand before breakdown occurs^49^. This property is important, since it is common for a substrate to be subjected to high voltages during its operation in a hybrid module. In the present investigation, ZAT shows a dielectric strength of about 12.9 kV/mm, which is comparable with commercially available alumina HTCC tapes (10–15 kV/mm) supplied by Adtech, Kyocera, Marua, etc.^50^,^51^,^52^ (see Table 2).

The microwave dielectric properties of ZAT and alumina tape are shown in Table 3. It is evident that the green tapes show low permittivity and high dielectric loss owing to the presence of organic additives such as dispersant, plasticizer, binder etc. During thermo-lamination, some of the polymers present in it bond well with the filler particles, making the laminated tape denser. As a result, a marginal decrease in dielectric loss with a slight increase in dielectric constant are observed^20^. After firing, the improvement in dielectric constant and a remarkable decrease in dielectric loss are mainly due to the densification of sintered tape which occurs by the combined effect of burnout of organic additives, removal of pores, microstructural grain growth and the strong bonding of ceramic particles during sintering^53^. The sintered ZAT tape shows good microwave dielectric properties with ε_r_ of 9.6 and tan δ of 8.4 × 10^-4^ at 5 GHz. The microwave dielectric properties of sintered alumina tape shows ε_r_ of 9.2 and tan δ of 9.1 × 10^−3^ at 5 GHz.

Comparison of commercial HTCC alumina substrate with NIIST ZAT substrate is shown in Table 2. Scanning through the table, one can conclude that the newly-developed ZAT substrate possesses comparable or even better characteristics in reference to alumina. The thermal characteristics such as thermal conductivity and thermal expansivity are comparable to alumina. Furthermore, the substrate possesses a unique thermal stability of dielectric constant, a property that most of the commercial ceramic substrates could not meet.

## Method

### Preparation of ZAT and alumina ceramics

The 0.83ZnAl_2_O_4_-0.17TiO_2_ ceramics were prepared by solid-state ceramic route using high purity ZnO (99.9% Sigma - Aldrich), Al_2_O_3_ (99.7% Sigma - Aldrich) and anatase TiO_2_ (99.8% Sigma - Aldrich) as the starting materials. The stoichiometric amounts of ZnO and Al_2_O_3_ were mixed and ball-milled using zirconia balls in ethanol medium for 24 h. The resultant slurry was dried and calcined at 1150 °C for 4 h to form ZnAl_2_O_4_. Then the mixture was ball milled with anatase TiO_2_ for 24 h and dried. The finely crushed powder was mixed with 5 wt.% polyvinyl alcohol binder and then pelletized into cylindrical pucks (8–15 mm diameter and thickness 2–8 mm) under a uniaxial pressure of 150 MPa. These green pellets were sintered at 1550 °C for 4 h at a rate of 5 °C/min. Alternatively, alumina (Al_2_O_3_) ceramic pucks were also synthesized from Al_2_O_3_ (99.7% Sigma - Aldrich), after ball milling, sieving, forming and sintering at 1600 °C for 4 h. The densities of the sintered pellets were measured using Archimedes method. To characterize the phase formation, the bulk ceramics were analyzed by X-ray diffraction technique using Cu K_α_ radiation (Philips X’Pert PRO MPD X-ray diffractometer, Philips, Almelo, Netherlands). The average particle size of ZAT and alumina samples were estimated through dynamic light scattering (ZetasizerNanoSeries:ZEN 3600, Malven, Worcestershire, UK) after dispersing the respective powder samples in distilled water and sonicated for 10 mins.

### Preparation of ZAT and alumina substrates through tape casting

To prepare ZAT tape casting slurry, a mixture of ethanol and xylene in the ratio of 50:50 (weight %) was used as the solvent system while fish oil as the dispersant (Arjuna Natural Extracts, Kerala, India). Initially, dispersion stability study of the slip was carried out by systematically varying the concentration of the dispersant with fixed ceramic powder loading. The shear viscosity of the slurry was measured using a rheometer (Brookfield, R/S Plus, MA, USA). In first stage of the slurry preparation, ZAT powder was ball milled for 24 h with the solvents (xylene/ethanol), besides fish oil (2.5 wt. % with respect to the powder). In the second stage, other essential vehicle components such as plasticizers, binder and homogenizer were added and ball milled for another 24 h. In the present formulation, benzyl butyl phthalate (BBP) (Sigma–Aldrich), polyethylene glycol (PEG) (Sigma Aldrich), polyvinyl butyral (PVB) (Butvar B-98, Sigma–Aldrich) and cyclohexanone (Sigma–Aldrich) were respectively used as Type I plasticizer, Type II plasticizer, binder and homogenizer^7^. The final slurry composition was then de-aired in a vacuum desiccator for 5 min to remove any entrapped air bubbles. Tape casting was done using a laboratory casting machine (Keko equipment, Žužemberk, Slovenia) and casting was carried out on a Mylar^®^ film using a doctor blade system. The casted tape was allowed to dry at room temperature for 12 h. After drying at room temperature, the thickness, dielectric properties of single layer green tape was measured and the TGA was performed using a thermo gravimetric analyzer (PerkinElmer, Waltham, USA). For further characterization, the 8 layer of green tape was cut into 40 mm × 40 mm square pieces and were laminated together. The lamination was done in an isostatic lamination press with a temperature of 70 °C for 20 min and pressure of 6 MPa (ILS-46, Haikutech, The Netherlands). The laminated tape was sintered at 1500 °C for 2 h. Alumina substrate was prepared in same way as reported by Mistler^7^ and green tape was sintered at 1600 °C for 2 h.

### Characterization of substrates

The sintered tapes were appropriately cut into square wafers (30 × 30 mm) with the help of an automatic cut-off machine (Discotom 100, Streurs A/s, Ballerup, Denmark) and polished using an automatic grinding and polishing machine (Tegramin-25, Streurs A/s, Ballerup, Denmark). The grinding and polishing were done in various stages using different composite discs such as MD Allergo, MD Piano, MD DAC and MD Chem for fine grinding, polishing and final polishing of all materials. Along with the discs, a diamond suspension and a standard colloidal silica suspension were used for lubrication and fine polishing. The microstructure of ZAT ceramic tape was studied using scanning electron microscopy (JEOL-JSM 5600 LV, Tokyo, Japan). The surface roughness of the sintered tape was observed using an atomic force microscope (AFM) (Bruker Nano Inc., USA) operating in the tapping mode regime. In order to investigate the mechanical properties of ceramic tape, nanoindentation testing was done using Hysitron TI 950 TriboIndenter with north star cube-corner diamond indenter tip (centerline-to-face angles of 35.3°) and with an *in-situ* SPM imaging using closed loop scanner. The CTE of ZAT ceramics sintered at 1550 °C and Al_2_O_3_ ceramics sintered at 1600 °C were measured by a thermo mechanical analyzer (TMA SS7300, SII Nano Technology Inc). The thermal conductivity of both alumina and ZAT were assessed by laser flash thermal properties analyzer (Flash Line 2000, Anter Corporation, Pittsburgh, USA). To find the dielectric strength of the ZAT tape, the voltage-current characteristics was calculated with the aid of Keithely 2410 (1100 V) source meter using a series of specimen within the thickness range of 50–60 μm. The dielectric properties up to 3 MHz are measured using LCR meter (HIOKI 3532–50 LCR Hi TESTER, Japan). The microwave dielectric properties of polished ceramic substrates were measured in a split post dielectric resonator (SPDR) operating at 5.155 GHz with the help of the vector network analyzer (8753ET, Agilent Technologies, Santa Clara, CA).

### Summary

To summarize, low dielectric loss ceramic tapes based on 0.83ZnAl_2_O_4_ -0.17TiO_2_ (ZAT) were developed using organic tape casting technique, and their microstructural, thermal, dielectric and mechanical properties were evaluated, in comparison to alumina substrates synthesized through a similar technique. ZAT substrates show an average CTE value of about 6.59 ppmK^−1^, which is compatible with the CTE values of the semiconductor devices embedded in electronic circuits and possess a relatively high thermal conductivity of 31.3 Wm^−1^K^−1^ at room temperature. The microwave dielectric properties of this substrate material (ε_r_ = 9.6 and tanδ = 8.4 × 10^−4^ at 5 GHz) are comparable to that of alumina, while *τ*_*f*_ is + 3.9 ppmK^−1^, which is close to zero, a feature that cannot be met with any of the alumina-based HTCC substrates. In automotive, avionics and space applications where the thermal stability of dielectric constant of substrates matter, the development of a new type of substrate with promising thermal and dielectric properties can be beneficial.

## Additional Information

**How to cite this article**: Roshni, S. B. *et al*. Can zinc aluminate-titania composite be an alternative for alumina as microelectronic substrate? *Sci. Rep.* **7**, 40839; doi: 10.1038/srep40839 (2017).

**Publisher's note:** Springer Nature remains neutral with regard to jurisdictional claims in published maps and institutional affiliations.

## Supplementary Material

Supplementary Information

## Figures and Tables

**Table 1:** Composition of ZAT and alumina tape casting slurry.

Component	Composition of ZAT (wt. %)	Composition of alumina (wt. %)	Function
**First stage**			
	74.8 (0.83ZnAl_2_O_4_−0.17TiO_2_)	61.94 (Aluminium oxide)	Ceramic powder
Fish oil	1.9	1.24	Dispersant
Xylene	9.4	15.31	Solvent
Ethanol	9.4	15.31	Solvent
**Second stage**			
Polyvinyl butyral	2.2	3.09	Binder
Benzyl butyl phthalate	1.1	1.55	Plasticizer (Type I)
Polyethylene glycol	1.1	1.55	Plasticizer (Type II)
Cyclohexanone	0.1	3.09	Homogenizer

**Table 2:** Comparison of commercial HTCC alumina substrate with NIIST ZAT.

Supplier	Product	Dielectric Constant ε_r_	tan δ (x10^−4^)	CTE (ppmK^−1^)	Thermal Conductivity (Wm^−1^K^−1^)	Dielectric Strength kV/mm	Reference
Kyocera	A-473 93% Al_2_O_3_	8.8 (1 MHz)	6	7.1	22	12	51
	A-476 96% Al_2_O_3_	9.4 (1 MHz)	4	7.2	27	12	51
Maruwa	Al_2_O_3_ (92%)	9.0 (1 GHz)	—	7	16	10	52
Coorstek	Al_2_O_3_ (96%)	9.8 (1 MHz)	1	7	25.5	22.6	54
Adtech	Al_2_O_3_ (92%)	9.2 (10 GHz)	30	6.57	20.3	11.6	50
NTK	Al_2_O_3_ (HA-921)	8.8 (10 GHz)	10	6.8	17	15	55
SCHOTT	Al_2_O_3_ ( > 92%)	9.0 (10 GHz)	10	6.8	17	—	56
NIIST	Al_2_O_3_	9.2 (5 GHz)	91	6.76	24.5	—	Present work
NIIST	ZAT	9.6 (5 GHz)	8.4	6.59	31.3	12.9	Present work

**Table 3:** Microwave dielectric properties of ZAT and alumina tape.

	Material	No. of layers	Thickness (mm)	Relative Density (%)	Dielectric properties at 5 GHz	
					**ε**_**r**_	**tan δ**
ZAT	Green Tape	1	0.21	56.7	5.6	9.7 × 10^−2^
	Laminated stack	8	1.48	70.0	6.3	6.7 × 10^−2^
	Sintered stack	8	1.26	97.5	9.6	8.4 × 10^−4^
Alumina	Green Tape	1	0.15	57.5	4.5	5.3 × 10^−2^
	Laminated stack	8	1.45	58.0	5.4	5.2 × 10^−2^
	Sintered stack	8	1.05	94.8	9.2	9.1 × 10^−3^
