# Supplementary material for: Can zinc aluminate-titania composite be an alternative for alumina as microelectronic substrate?
Source: Sci Rep. 2017 Jan 13;7:40839. doi: 10.1038/srep40839 (PMC5234029; doi:10.1038/srep40839)
Supplement: Supplementary Information [file srep40839-s1.pdf]

Zinc aluminate-titania composites outperform alumina as microelectronic substrates

Satheesh Babu Roshni[a], Mailadil Thomas Sebastian[b], Kuzhichalil Peethambharan Surendran[a]*

[a]Materials Science and Technology Division,

[a]National Institute for Interdisciplinary Science and Technology, CSIR, Trivandrum 695019, India

[b]Microelectronics Research Unit, Faculty of Information Technology and Electrical Engineering, University of Oulu, 90014 Finland

*Corresponding Author; Email: kpsurendran@niist.res.in

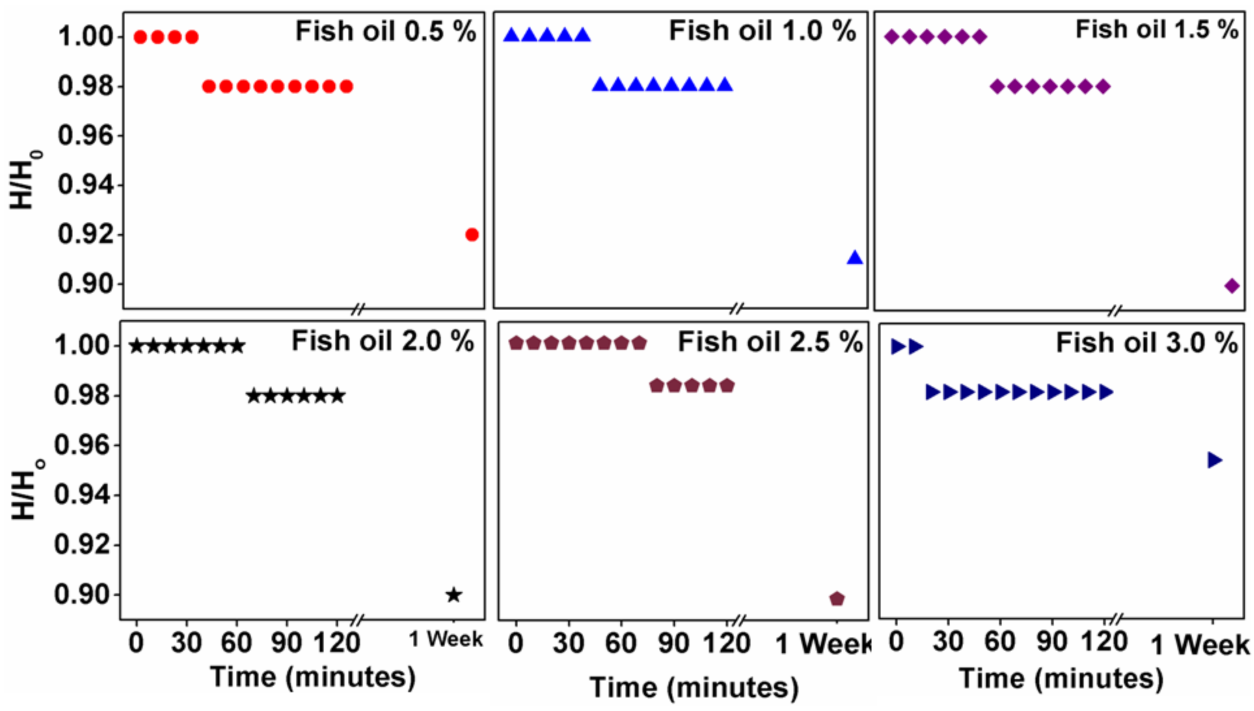

Supplementary Fig. S1 Sedimentation analysis of ZAT slurry at different concentration of fish oil (dispersant)

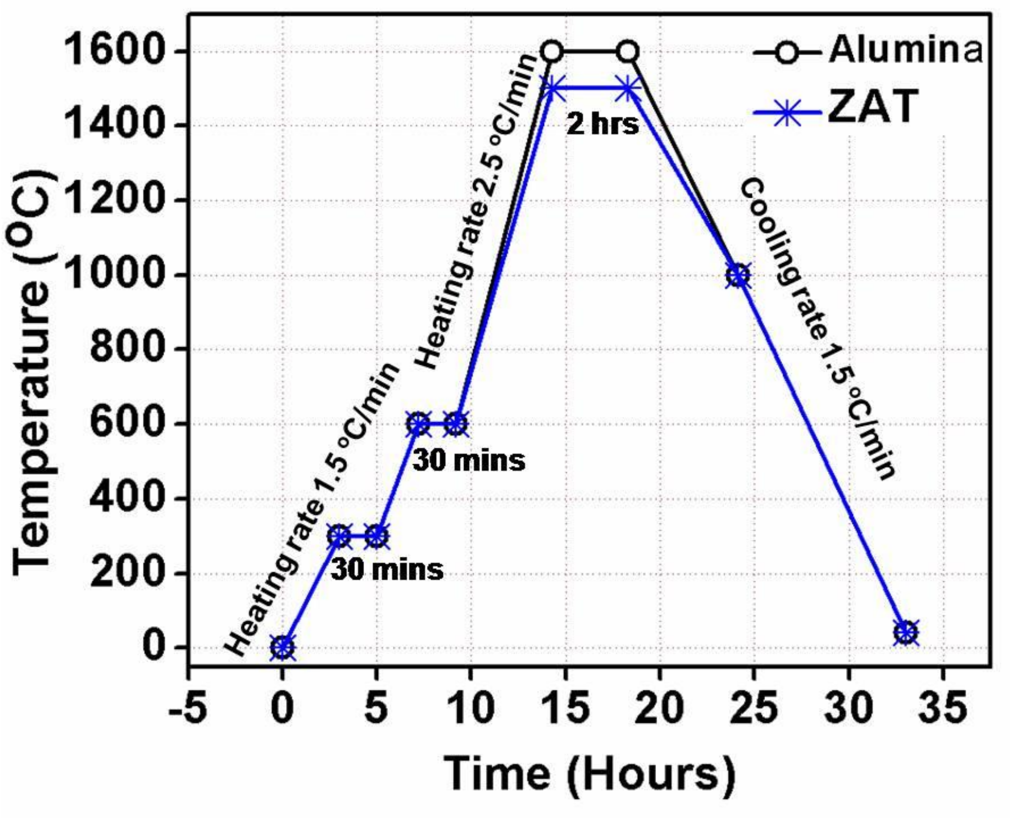

Supplementary Fig. S2 Sintering profile of ZAT and alumina tape

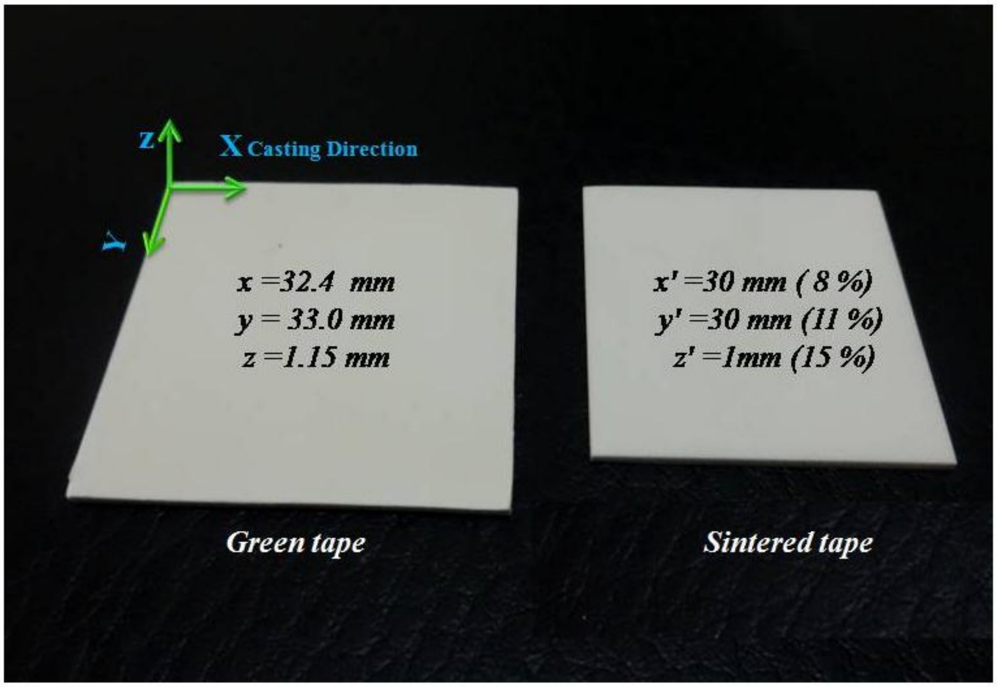

Supplementary Fig. S3 Photograph showing the actual shrinkage of ZAT tape during sintering

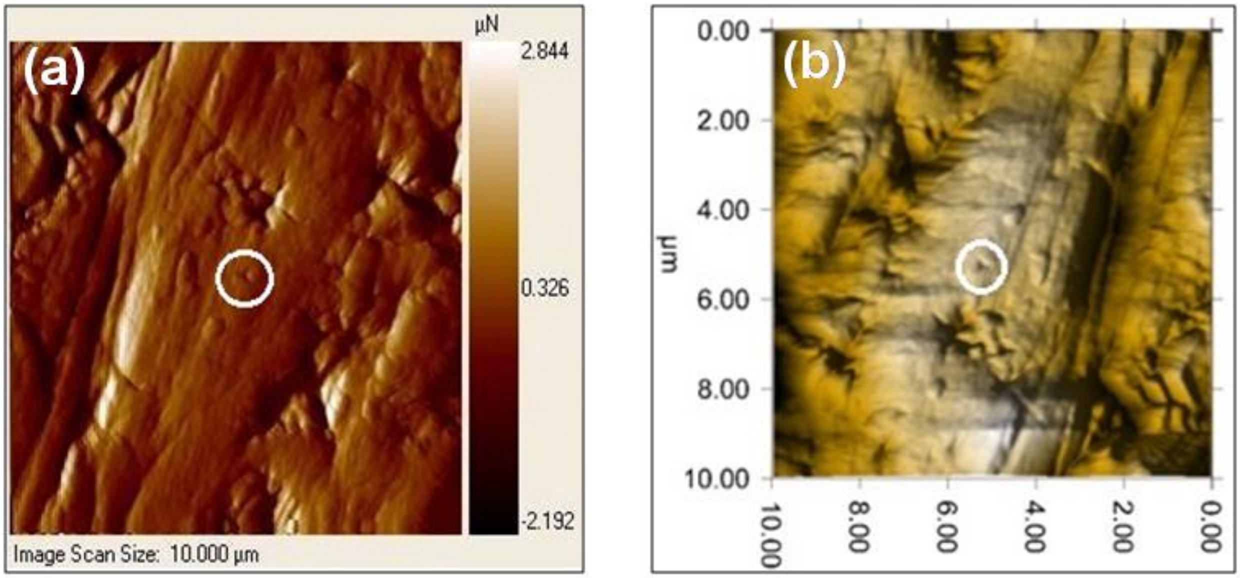

Supplementary Fig. S4 (a) Gradient and (b) 3D in situ scanning probe microscopic (SPM) image of ZAT after 1500 µN indentation
